# Supplementary material for: An Exploration of Small Molecules That Bind Human Single-Stranded DNA Binding Protein 1
Source: Biology (Basel). 2023 Nov 6;12(11):1405. doi: 10.3390/biology12111405 (PMC10669474; doi:10.3390/biology12111405)
Supplement: Supplementary file 1 [file biology-12-01405-s001.zip › biology-2660561-supplementary.pdf]

Supporting Information

An Exploration of Small Molecules that Bind Human Single-Stranded DNA Binding Protein 1

Zachariah P. Schuurs [1,2], Alexander P. Martyn [1,2], Carl P. Soltau [3], Sam Beard [2], Esha T. Shah [2,4], Mark N. Adams [2,4], Laura V. Croft [2,4], Kenneth J. O'Byrne [2,5], Derek J. Richard [2,4] and Neha S. Gandhi [1,2,6,*]

[1] Centre for Genomics and Personalised Health, School of Chemistry and Physics, Queensland University of Technology (QUT), Brisbane, QLD 4000, Australia; zachariah.schuurs@hdr.qut.edu.au

[2] Cancer and Ageing Research Program, Centre for Genomics and Personalised Health, Queensland University of Technology (QUT), Translational Research Institute (TRI), Woolloongabba, QLD 4102, Australia; alexander.martyn@qut.edu.au (A.P.M.); sam.beard1@gmail.com (S.B.); mn.adams@qut.edu.au (M.N.A.); laura.croft@qut.edu.au (L.V.C.); k.obyrne@qut.edu.au (K.J.O.); derek.richard@qut.edu.au (D.J.R.)

[3] School of Chemistry and Physics, Centre for Materials Science, Queensland University of Technology,
Brisbane, QLD 4000, Australia; c.soltau@qut.edu.au

[4] School of Biomedical Sciences, Faculty of Health, Queensland University of Technology, Translational
Research Institute, Woolloongabba, QLD 4102, Australia

[5] Cancer Services, Princess Alexandra Hospital—Metro South Health, Woolloongabba, QLD 4102, Australia

[6] Department of Computer Science and Engineering, Manipal Institute of Technology, Manipal Academy of Higher Education, 576104, Manipal, India

* Correspondence: neha.gandhi@qut.edu.au or neha.gandhi@manipal.edu

1.1 SUPPLEMENTARY METHODS

1.1.1 Design of the AlphaLISA screening assay

To test whether the compounds bind to hSSB1 and to design an effective screening assay, we used AlphaLISA technology from Perkin Elmer. This technology involves binding a biotin-labelled control DNA oligonucleotide (btn-In3-PD) to a streptavidin-coated donor (SA-donor) bead. hSSB1 is bound to an anti-hSSB1 sheep antibody as described previously [1–3], which binds to Protein G-coated acceptor beads. When hSSB1 and btn-In3-PD bind, they bring the two beads close together, and with excitation at 680 nm by laser irradiation, the phthalocyanine in the donor bead excites ambient oxygen to a reactive form of O_2. When this is within 200 nm of a europium-containing acceptor bead (during a binding event), the reactive oxygen is transferred to the thioxene derivative, producing light at 615 nm. Without the acceptor bead, oxygen returns to its ground state. This lends the assay to a competition-based format for high-throughput screens.

A titration matrix is required to optimise assay conditions to develop an AlphaLISA assay that accurately acquires results and subsequently follows the Cheng-Prusoff equation to report IC50's as a molecule Kd. The main requirement of this is a high signal-to-background (S/B) ratio for the condition with the lowest concentration of analyte. This is determined by finding the hook point of signal, which indicates the highest signal before the bead is saturated, after which a decrease in signal is observed.

A series of titration matrix binding studies were performed to establish the optimal conditions for the assay. The initial experiment tested btn-In3-PD and hSSB1 from 500 to 0.05 nM with a 10-fold dilution factor, and each series was tested with Ab concentrations of 1, 3, and 10 nM. Under these conditions, the hook point was found at 5 nM hSSB1 and 50 nM of oligonucleotide. The matrix test was repeated with hSSB1 concentrations ranging from 5 to 0.3125 nM, and the btn-In3-PD concentrations ranging from 10 to 0.625 nM both with a dilution factor of 2-fold. These conditions were tested with antibody concentrations of 0.3 nM, 1 nM and 3 nM. From this we ascertained that with 1 nM of Ab, the optimal S/B ratio was with 0.5 nM hSSB1 and 5 nM of biotinylated oligonucleotide. The assay development tests had a Z' value of 0.77 and a signal-to-noise ratio of 101.

The order in which reagents are combined can significantly affect the signal. A range of mixing orders were tested with an in-house phosphorothiolated oligomer sequence (In3-PS) with the same sequence as the competitor In3-PD. In3-PS was added in a series of five-fold

serial dilution of concentrations from 1000 to 0.06 nM. The addition order and conditions with the optimal signal readout are described in the main text.

An immunoprecipitation assay was conducted to check that the compounds of interest did not interfere with the interaction between hSSB1 and the hSSB1 antibody used. In 150 µL of IGEPAL buffer, 1 µg of antibody was mixed with 10 µM of compound and 1 µg of hSSB1 protein. After incubation for 1 hour, 10 uL of Protein G beads (ThermoFisher) were added to each sample before being incubated for an additional 1 hour. The beads were washed with IGEPAL buffer. The beads were boiled with 2x loading buffer (2% β-mercaptoethanol) and run on an SDS-PAGE gel before staining with Coomassie Blue.

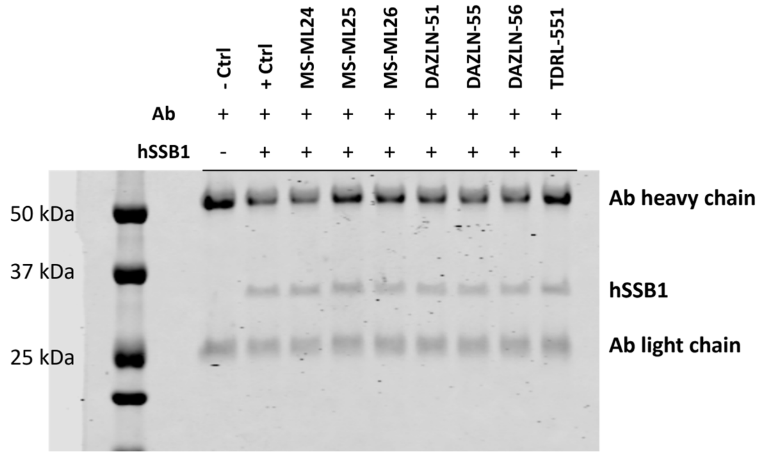

Figure S1: Immunoprecipitation of the antibody, hSSB1 and compound revealed that the compounds did not prevent the binding of hSSB1 to the antibody.

1.1.2 Simulation setup and visualization

Cosolvent molecular dynamics (MD) simulations were performed to predict the binding of hit compounds to hSSB1 and account for any conformational changes or the presence of allosteric sites in the protein. The benefit of cosolvent MD is that compared with classical MD, is the ligand is not placed in the binding pocket [4]. The hSSB1 monomer was isolated from PDB 4OWX and a single copy of the ligand was randomly placed at least 40 Å from the edge of the protein. The disordered C-terminal carboxyl tail of hSSB1 was not considered in the simulations [5]. Using LEaP from AmberTools version 21.3[6], the PBRadii was set to mbondi2, the small molecules were prepared using the GAFF2 forcefield [7], while ff19SB [8] forcefield was applied to the protein. The system was solvated in a truncated octahedral box with TIP3P [9] water extending 12 Å from the edge of the protein-ligand system. The system was neutralized with 0.15 M NaCl, with 56 Cl^- and 55 Na^+ ions placed using the tleap module of AMBER. The particle-mesh Ewald (PME) method [10] was used to treat long-range

electrostatics, and periodic boundary conditions (PBC) were applied during the simulations. The non-bonded cut-off was 12 Å. Five simulations for each compound were prepared in this manner, each with the compound in a different starting position. The MD simulation was performed multiple times, starting from different initial configurations of ligands, to assess the convergence of the results. Using AMBER2020 [6,11], each system first had the water minimized; then, the water was allowed to move, minimizing the system (1000 cycles), before the system was heated (300 K), and then relaxed (100ps). The system was equilibrated for 500 ps. The final production was performed using pmemd.cuda [12] and an NPT ensemble [6]. It was run for 300 ns, remaining at 300 K, with a snapshot recorded every 5000 steps.

1.1.3 Analysis of the trajectories

The trajectories were processed using the AMBER module CPPTRAJ [13] for centring, fixing hSSB1 within the periodic boundaries, and stripping the solvent. CPPTRAJ was again used to cluster the frames using *the k*-means algorithm into 10 clusters with a maximum of 500 iterations. The initial set of points was randomized and a random sieve of 10 was used to read the five trajectories. Clustering was used to determine the positions that the compound occupied the most during the simulation, and trajectories were output for each cluster, along with the top average and representative poses. Trajectories and clusters were visualized with ChimeraX version 1.4 [14,15], and interactions between the protein and bound compounds were visualized using Discovery Studio Visualizer version 21.1.0 [16].

1.1.4 Relative free binding energy calculations

The molecular mechanics Generalized Born surface area (MM/GBSA) [17] method was used on the trajectory outputs obtained from the clustering procedure to predict the relative free energy of binding and calculate the per-residue decomposition of the ligands to hSSB1 site. Only trajectories with the ligands bound to hSSB1 were considered. This prediction of the free energy of a ligand binding to a receptor to form a complex in a system is defined by the equation:

$$\Delta G_{binding} = \Delta G_{complex} - \Delta G_{receptor} - \Delta G_{ligand}$$

These energy components are broken down into contributions from the following components: bond, angle, dihedral, van der Waals, internal electrostatic component, polar component of solvation energy (according to the Generalized Born model), and non-polar component [18]. The AMBER19 implementation MMPBSA.py was used on the trajectory output from the top cluster of each ligand. The calculations were conducted starting from the

first production frame. GB methods were used to calculate the free energy at an ionic strength of 0.15 M, using igb=5, and the atomic radii (mbondi2) were calculated according to the topology files. The per-residue decomposition was conducted using the second scheme available in MMPBSA.py. The results were analysed and illustrated using GraphPad Prism version 9.4.1. Entropy of the interactions were not calculated as the purpose of the simulations was to locate the binding sites of the compounds.

1.2 SUPPLEMENTARY DATA

1.2.1 Comparison between hSSB1 and RPA

RPA is the only protein with published structures of inhibitors comparable to hSSB1. RPA and hSSB1 are not only structurally conserved, but there are aromatic residues in the ssDNA binding site of the RPA DBD-B that are conserved in the hSSB1 ssDNA-binding domain (**Figure S22**). The aromatic residues Trp-55 and Phe-78 (hSSB1) are conserved at residues 361 and 386, respectively, in the RPA DBD-B [19]. Aromatic residues in RPA DBD-A subunit are Trp-212, Phe-369 and Phe-238 and are not conserved in the same positions in hSSB1. There are other non-aromatic residues conserved between the ssDNA binding site: Thr-30 and Lys-31 (hSSB1) are conserved at positions 330/331 (RPA), Ser-76 (hSSB1) at 384 (RPA), Gly-80 (hSSB1) at 388, and Leu-84 (hSSB1) at 391 (RPA). The aromatic residues Tyr-74 and Tyr-85 in hSSB1 are not conserved in RPA. From the crystal structures of SOSS1 complexes, which contain hSSB1 bound to poly-T (PDB:4OWX [20]), the four aromatic residues in the ssDNA binding site (Trp-55, Phe-78, Tyr-74, and 85) are essential for facilitating the binding of ssDNA nucleotides through π–π stacking interactions. Similar roles of aromatic residues were observed in the RPA ssDNA binding site between DBD-A and DBD-B (PDB:1JMC [21]). Binding sites that share similar structures and conserved residues led to the hypothesis that the small molecules developed to inhibit RPA would similarly bind to hSSB1 and serve as a starting point for the development of small molecule inhibitors of hSSB1.

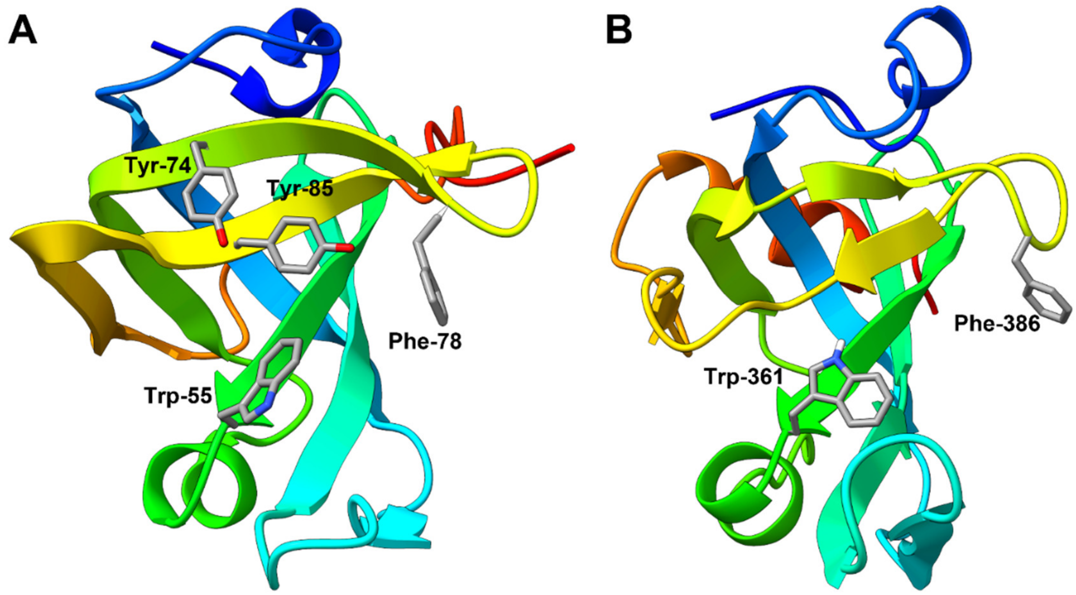

Figure S2: Conserved aromatic residues in the ssDNA binding site of hSSB1 (PDB: 4OWX) (B) and the DBD-B of RPA (PDB: 1JMC) which covers residues 301-422 (A). The standard structure of the OB-fold can be observed in both examples, with the five β-strands forming the barrel, and the α-helix between strands three and four. The proteins are coloured from their N-terminal (blue) to C-terminal (red) and residues are shown in sticks. These figures were rendered using ChimeraX version 1.4.

1.3 COMBINATORIAL LIBRARY DOCKING

Table S1: The compounds from the combinatorial library with the highest score when docked to hSSB1 with Cresset Flare™ [22,23], and the calculated LF Rank Score reported. The LF Rank Score is a prediction of the 3D protein-ligand complex. The H-bond details are calculated from SwissADME [24].

Rank	Structure	Molecular Weight	H-bond Acceptors	H-bond Donors	LF Rank Score
1	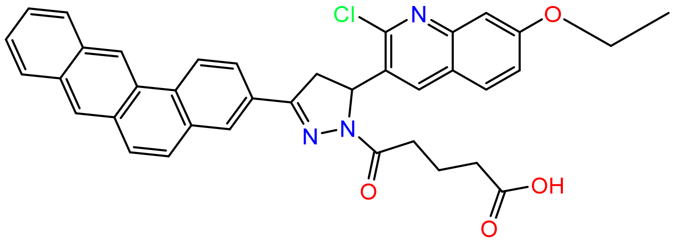	615.1	6	1	-11.31

2	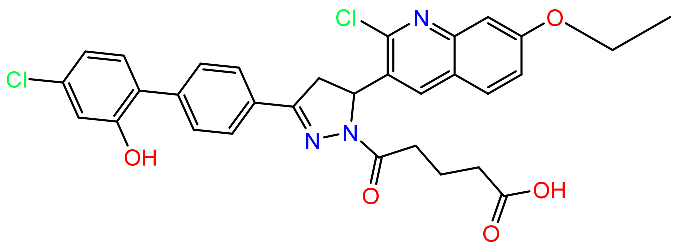	591.5	7	2	-11.29
3	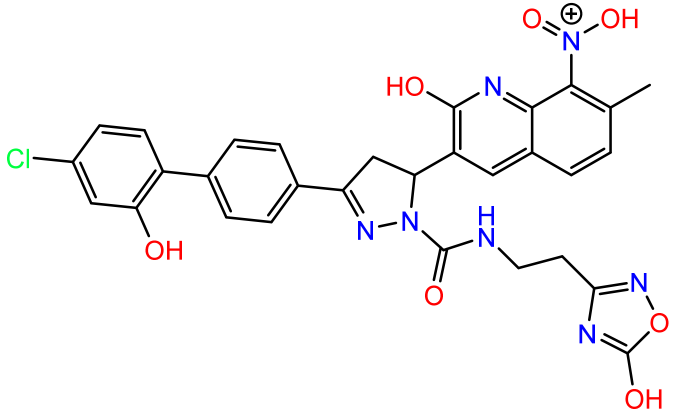	630.0	11	5	-11.16

4	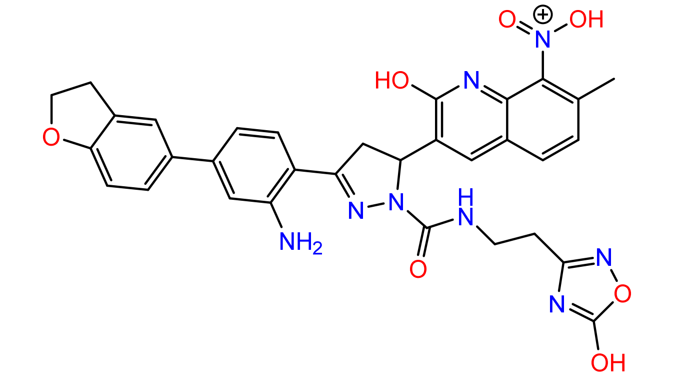	636.6	11	5	-10.73
5	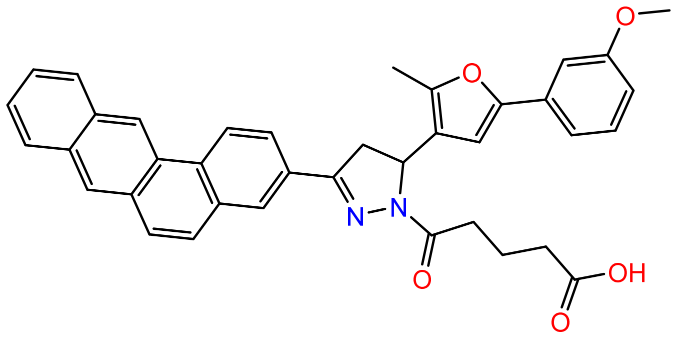	595.7	6	1	-10.68

6	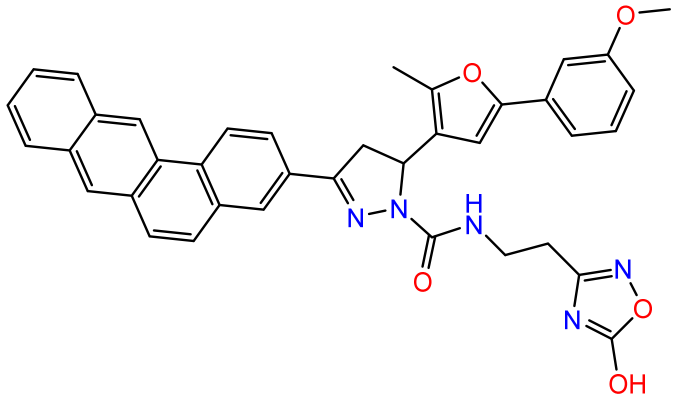	637.7	8	2	-10.68
7	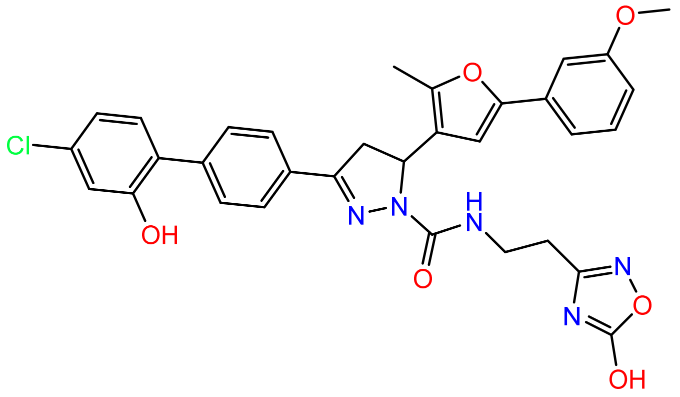	614.1	9	3	-10.55

8	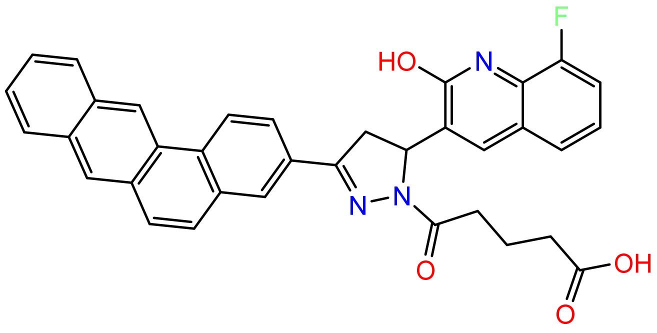	570.6	7	2	-10.55
9	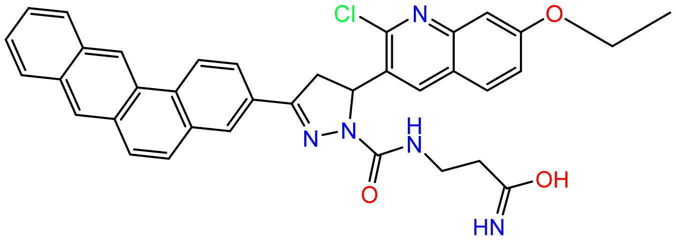	616.1	6	3	-10.54

10	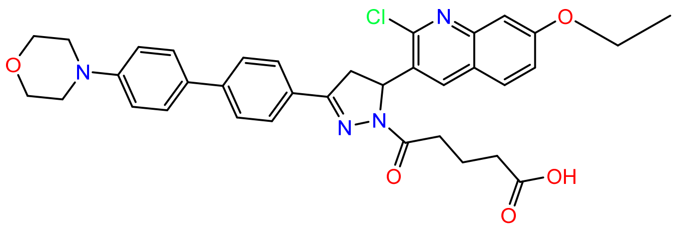	626.1	7	1	-10.46

1.4 COMPOUND SPECTRA

Nuclear magnetic resonance (NMR) was performed using a Bruker AVANCE III 600 MHz NMR Spectrometer at 25°C and chemical shifts are reported as δ (ppm) relative to the solvent used. High-resolution mass spectrometry (HRMS) was performed on a Thermo Scientific LTQ Orbitrap XL ETD Hybrid Ion Trap-Orbitrap Mass Spectrometer equipped with a heated electrospray ionization (ESI) source operating in positive ion mode at a mass resolution of 120,000 (at m/z 400). High-performance liquid chromatography (HPLC) was performed on a Thermo Scientific Dionex UltiMate 3000 RSLC using an Agilent Prep-C18 Scalar column (10 μ, 150 × 4.6 mm). The general method was a gradient elution of 0-30% ACN in H_2O over 15 mins at 40 °C with a flow rate of 0.8 mL/min monitoring UV absorbance at 254 nm.

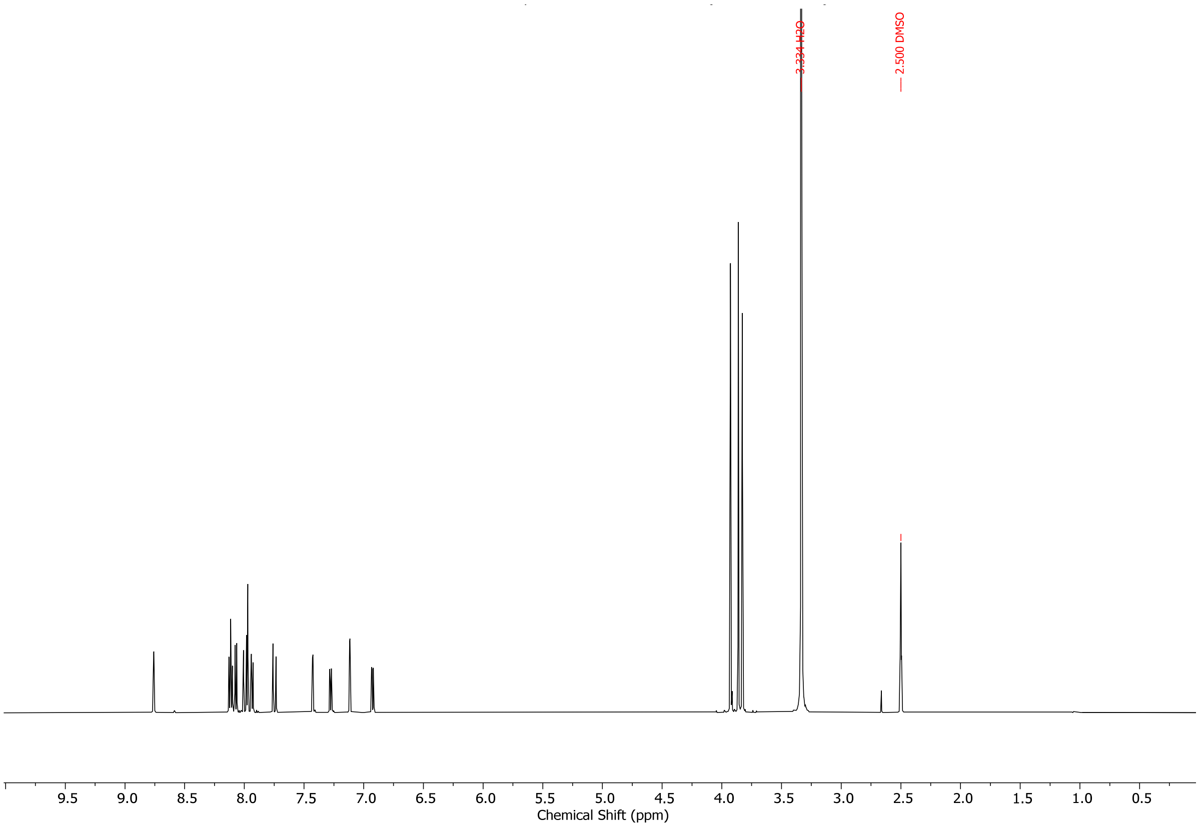

Figure S3: ^{1}H NMR (d6-DMSO, 600 MHz) of **3**.

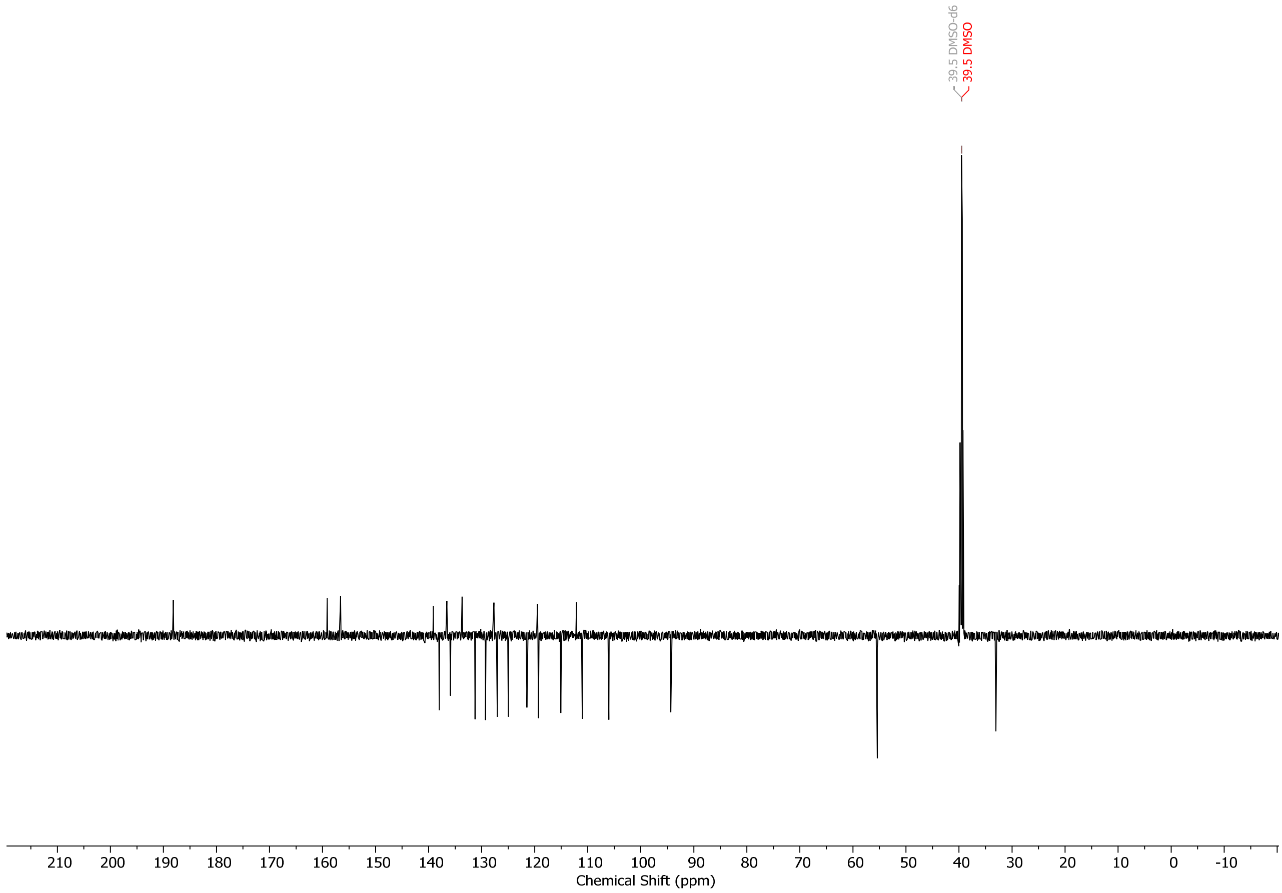

Figure S4: ^{13}C NMR (d6-DMSO, 150 MHz) of **3**.

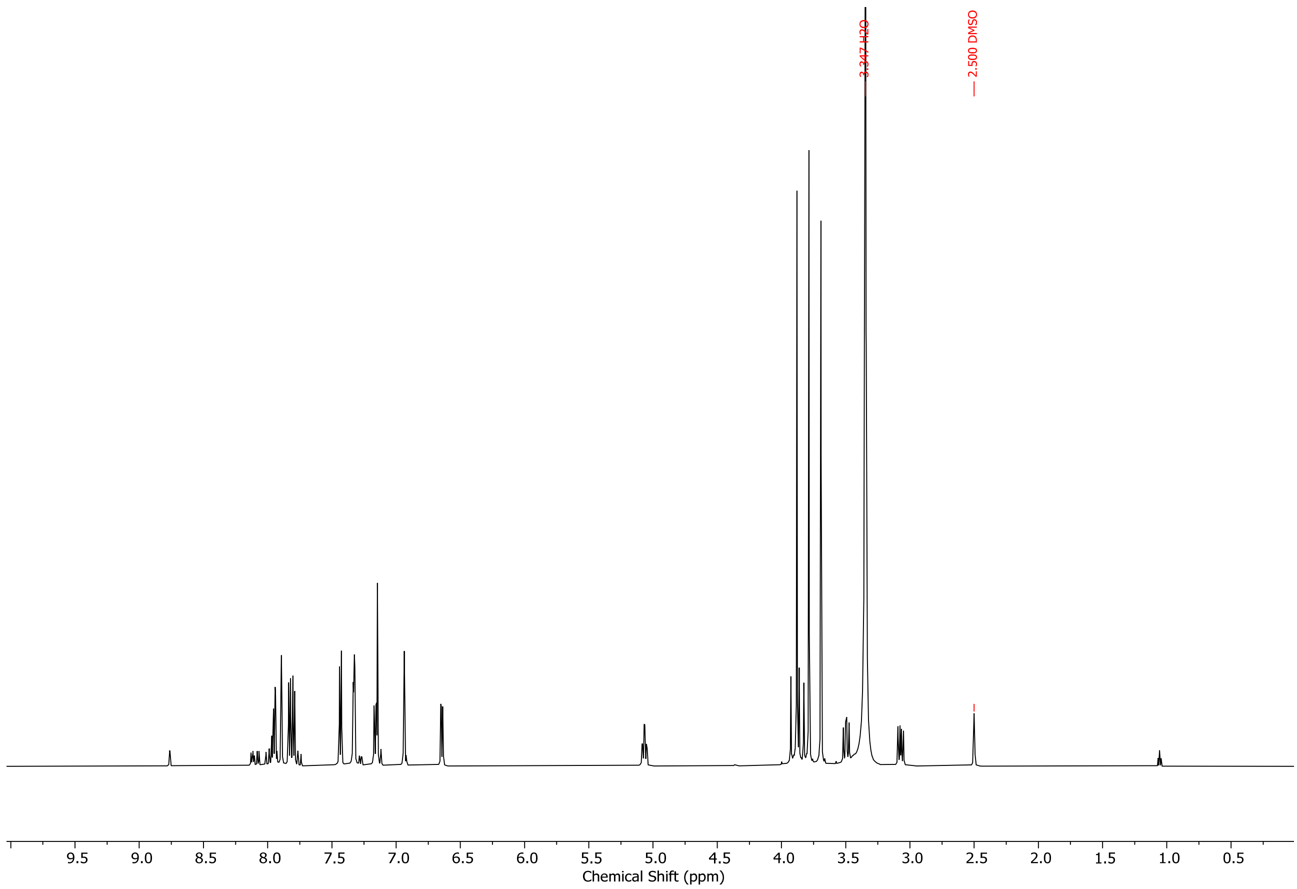

Figure S5: ^{1}H NMR (d6-DMSO, 600 MHz) of **4**.

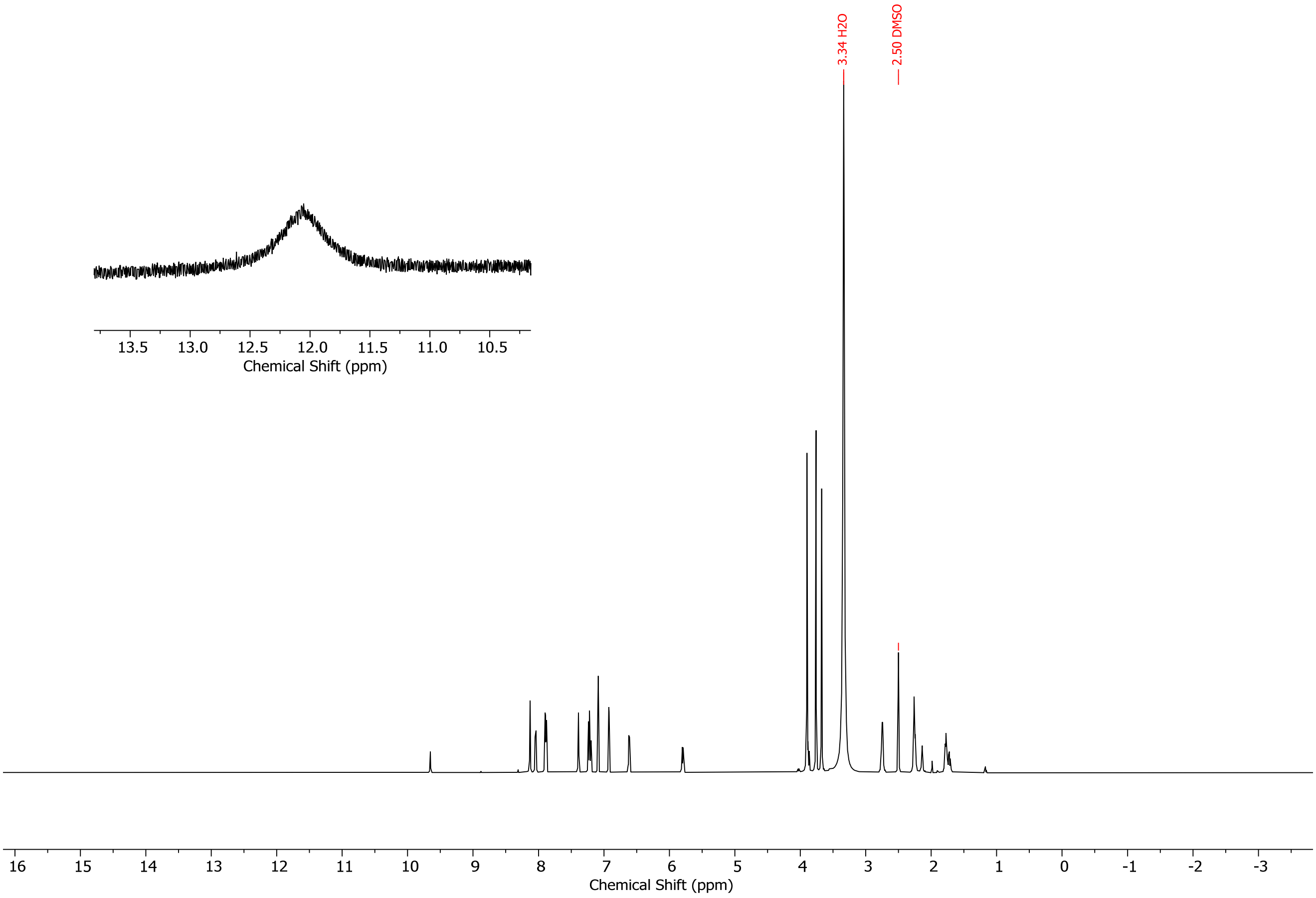

Figure S6: ^{1}H NMR (d_6-DMSO, 600 MHz) of **5**.

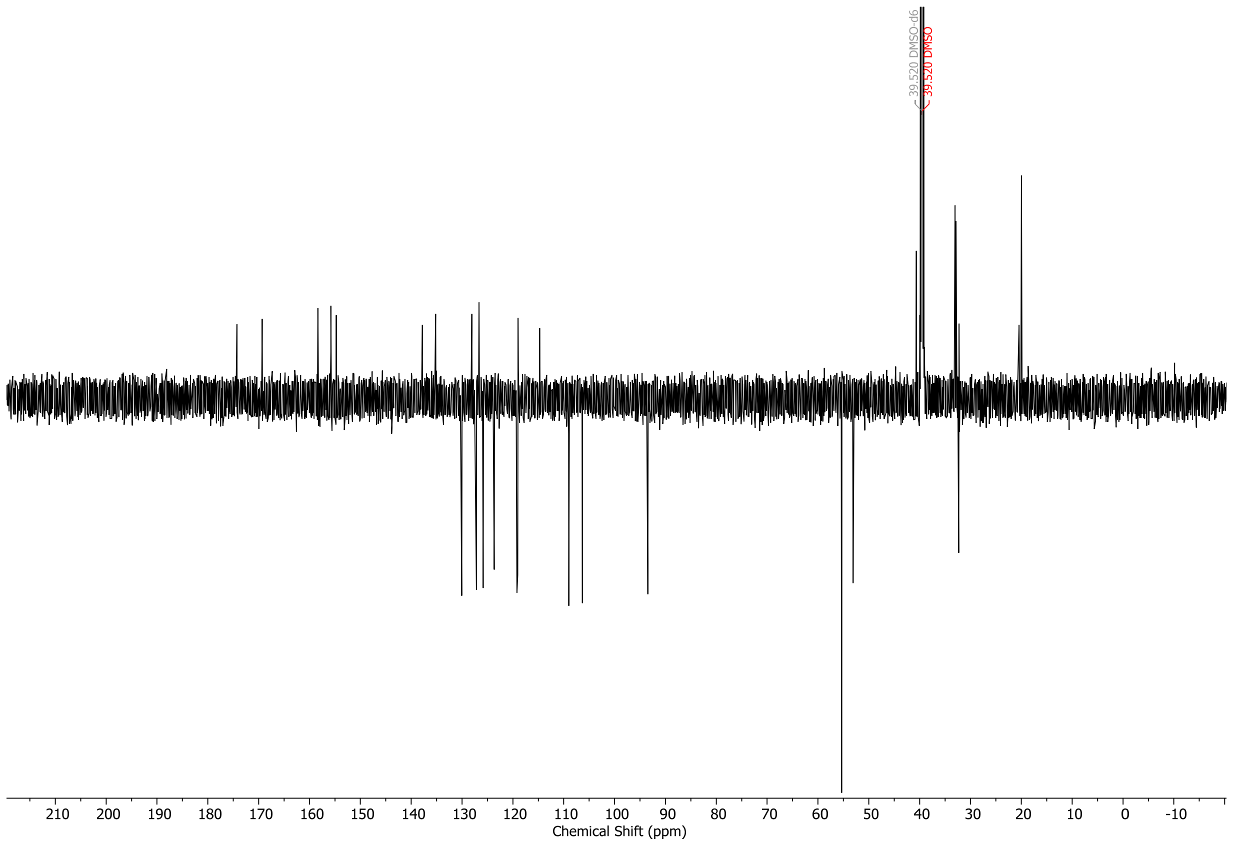

Figure S7: ^{13}C NMR (d_6-DMSO, 150 MHz) of **5**.

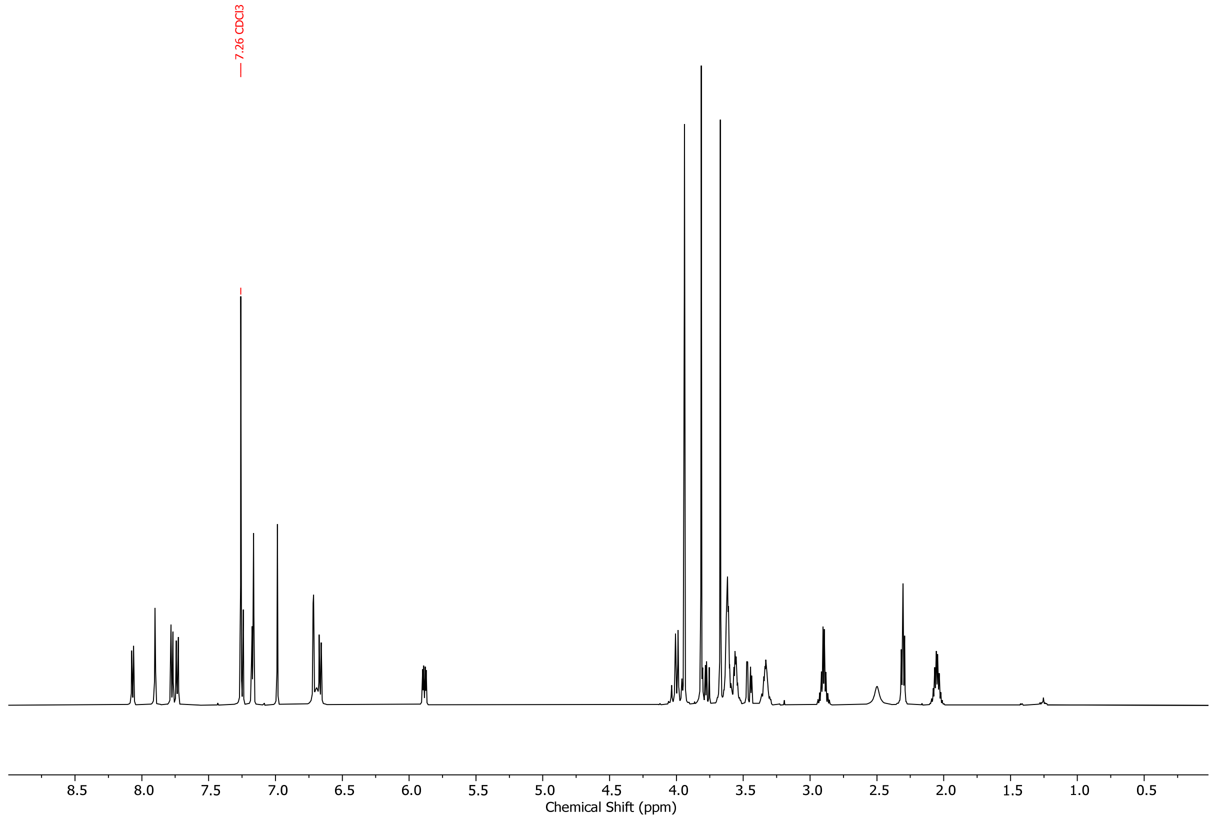

Figure S8: ^{1}H NMR (CDCl3, 600 MHz) of **6**.

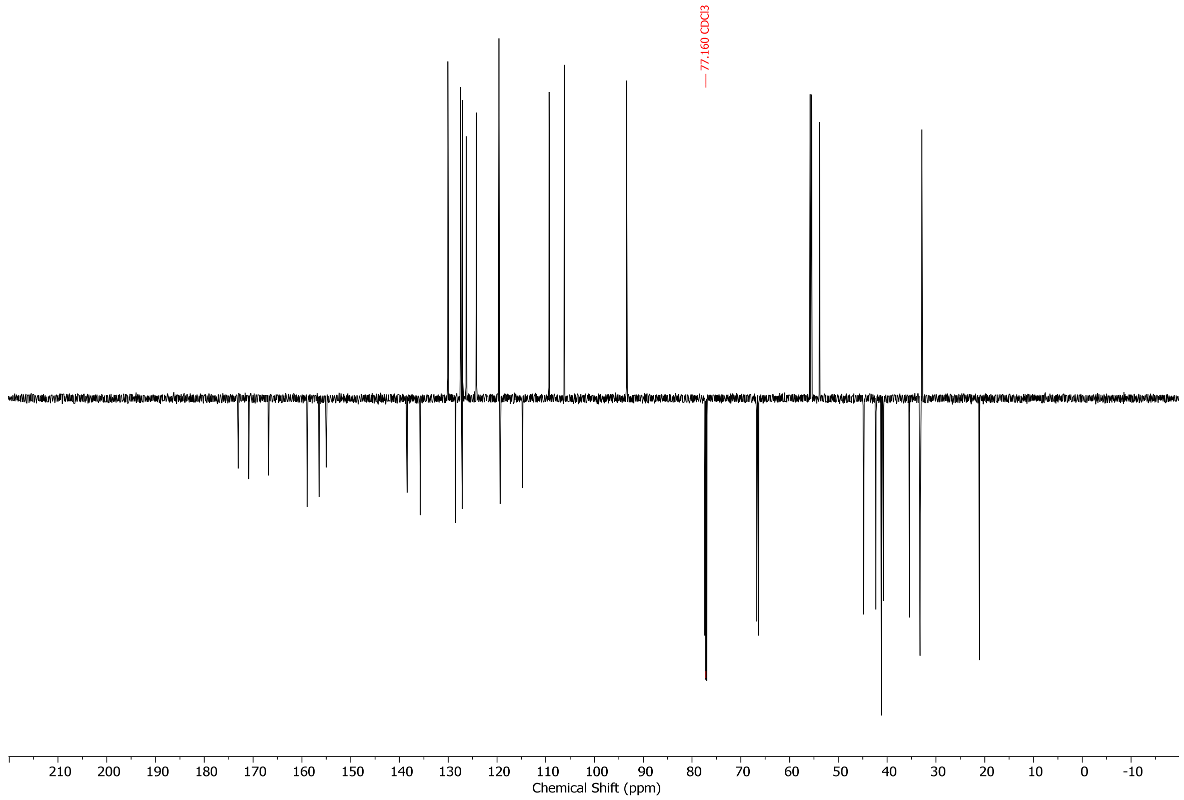

Figure S9: ^{13}C DEPTQ135 ($CDCl_3$, 150 MHz) of **6**.

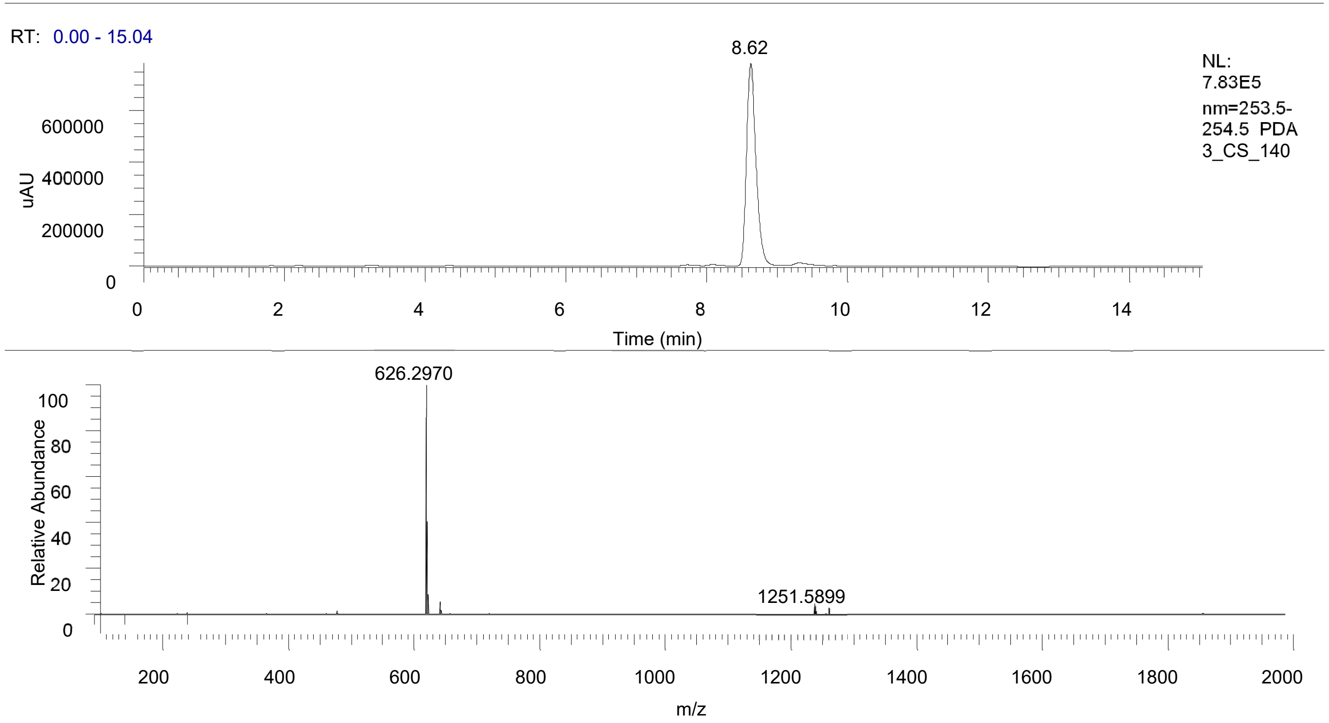

Figure S10: LC-MS analysis of **6**.

1.5 REPRESENTATIVE EMSA GELS

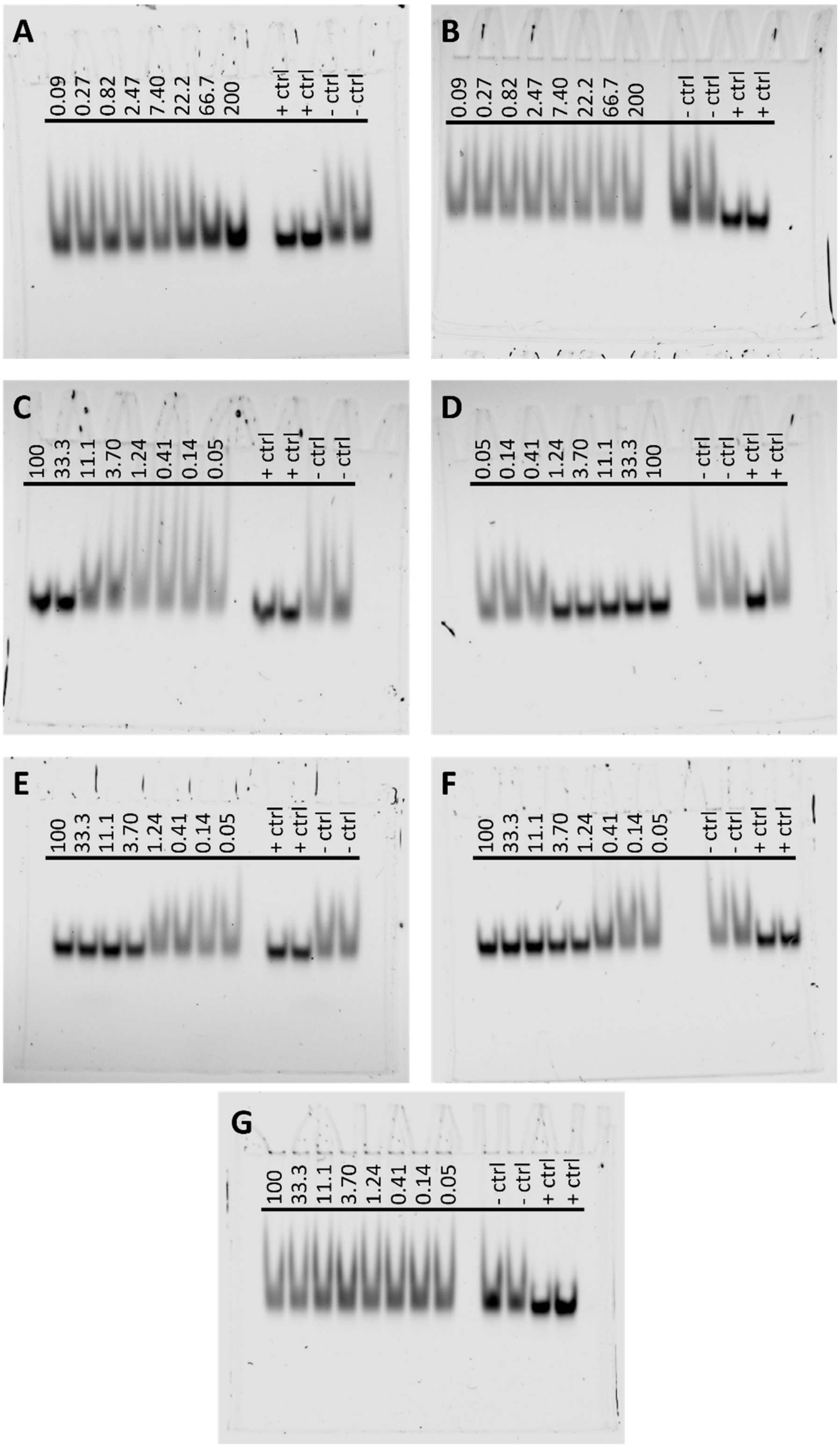

Figure S11: Representative EMSA gels of the compounds binding hSSB1. All concentrations are labelled in micromolar [µM]. The gels are in the order DAZLN-55 (A), DAZLN-56 (B), TDRL-551 (C), MS-ML24 (D), MS-ML25 (E), and MS-ML26 (F) and DAZLN-551 (G). The negative wells contained hSSB1 and Cy5-labelled oligonucleotide, while the positive control wells contained only Cy5-labelled oligonucleotide. Note that one of the positive control wells in (D) is erroneous, containing protein.

1.6 CLUSTERING OF COMPOUND OCCUPANCY

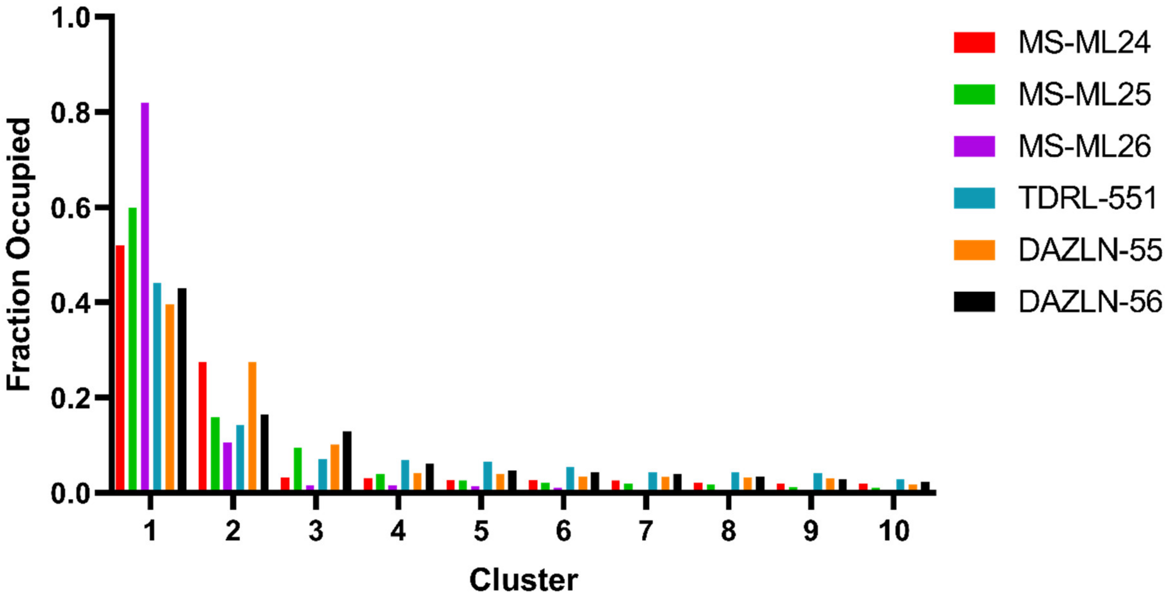

Figure S12: Fraction that each compound occupied a binding site of hSSB1 across all five trajectories, clustered using a k-means algorithm.

Table S2 The ΔG_{bind} (kcal/mol) for the top clusters of each compound, across the five trajectories. BS denotes the binding site, which is the site occupied by the compound in each of the highest clusters, where the compound was bound to the protein. Note that for BS2 of DAZLN-56, the compound was not bound to the protein, and was instead at a distance from the protein. The standard deviation (SD) is the reported value from the free energy calculations. This, and the other representative poses are presented in Figures S12-S13.

DAZLN-55			DAZLN-56			MS-ML24			MS-ML25			MS-ML26		
	Avg	SD		Avg	SD		Avg	SD		Avg	SD		Avg	SD
BS1	-23.32	±8.51	BS1	-25.1	±8.66	BS1	-30.79	±12.65	BS1	-26.91	±8.78	BS1	-27.88	±6.16
BS2	-16.39	±4.30	BS2	-8.15	±8.35	BS2	-23	±6.10	BS2	-23.03	±7.32	BS2	-17.76	±6.51
BS3	-10.18	±6.65	BS3	-16.84	±.60				BS3	-17.8	±5.68			
									BS4	-15.1	±7.02			

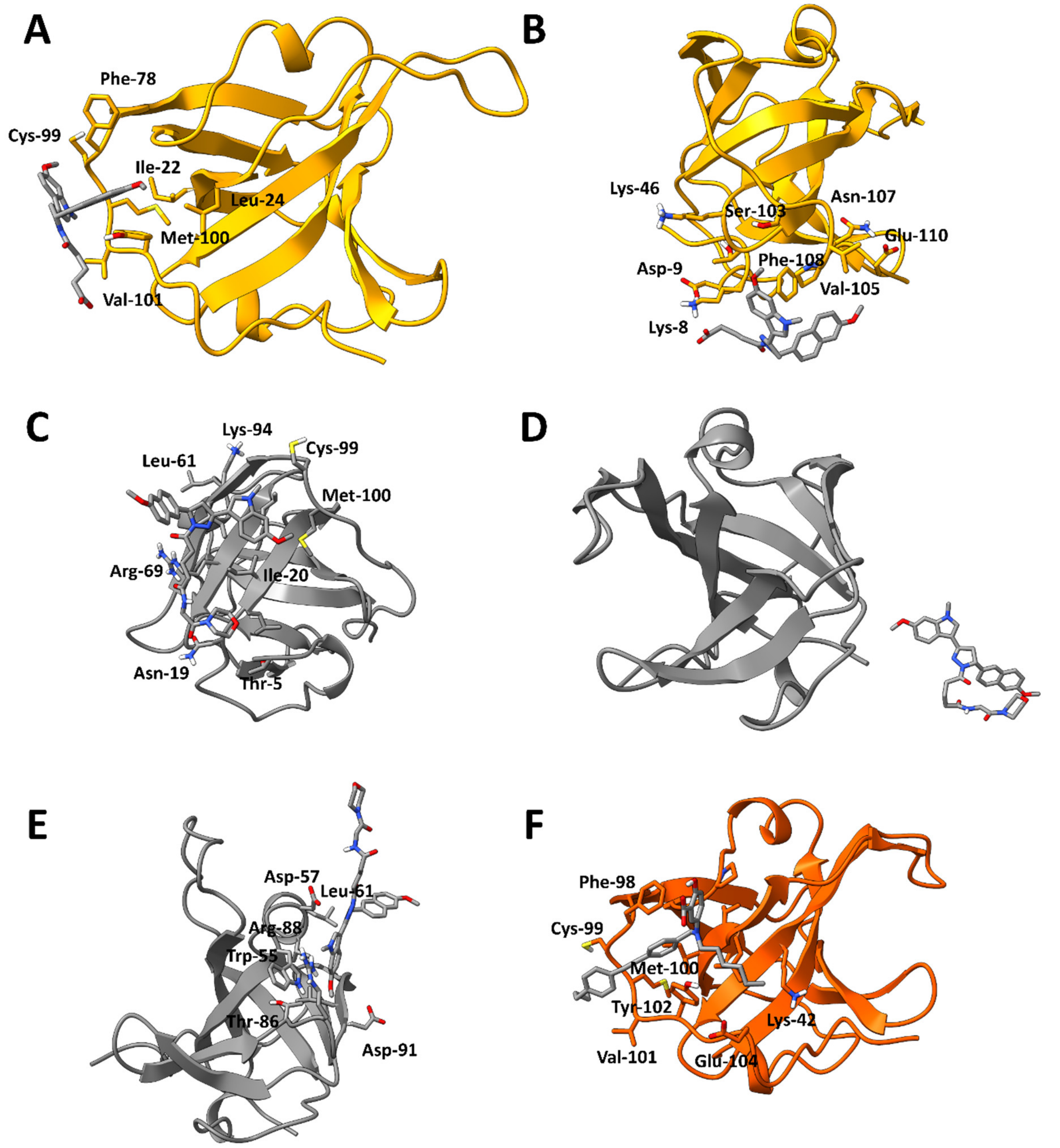

Figure S13: Representative poses of DAZLN-55 (A, B), DAZLN-56 (C,D,F) and MS-ML24 (F) bound to hSSB1 from the top clusters of the cosolvent MD simulations, rendered with ChimeraX version 1.4. A and B are BS2 and BS3 of DAZLN-55, while F is BS2 of MS-ML24. The ribbon structures are hSSB1; in all cases, the α-helix between the β-strands 3 and 4 is located at the top.

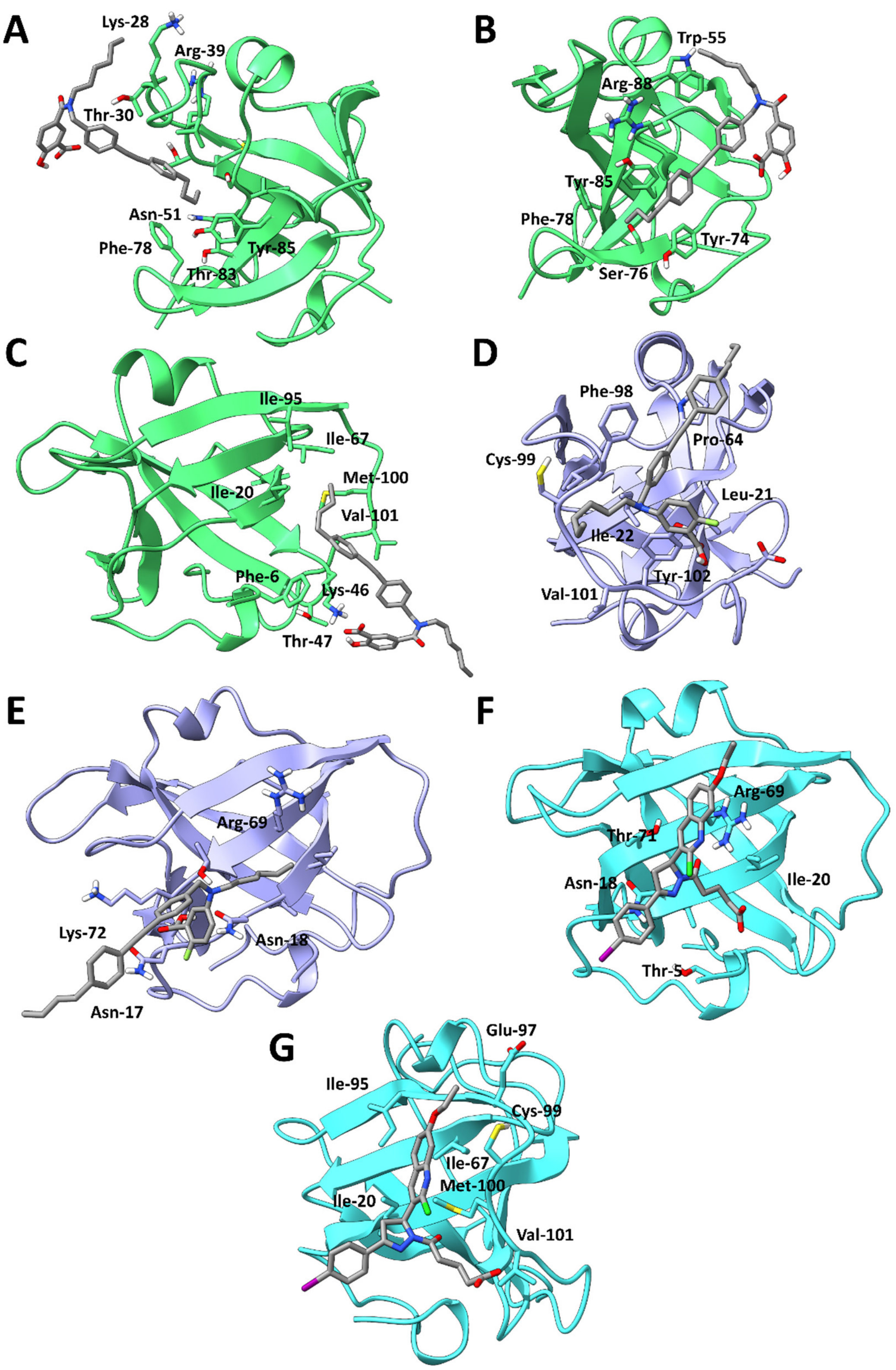

Figure S14: The representative poses for the clusters from the cosolvent MD simulations in where the compounds bound to hSSB1, rendered with ChimeraX version 1.4. A-C represent BS2-BS4 of MS-ML25; D and E are BS1 and BS2 of MS-ML26; and F and G are the representation of TDRL-551. The coloured ribbon structure represents hSSB1, and in each pose the α-helix between β-strands 3 and 4 are on the top of the representations.

1.7 INTERACTIONS OF TOP CLUSTERS

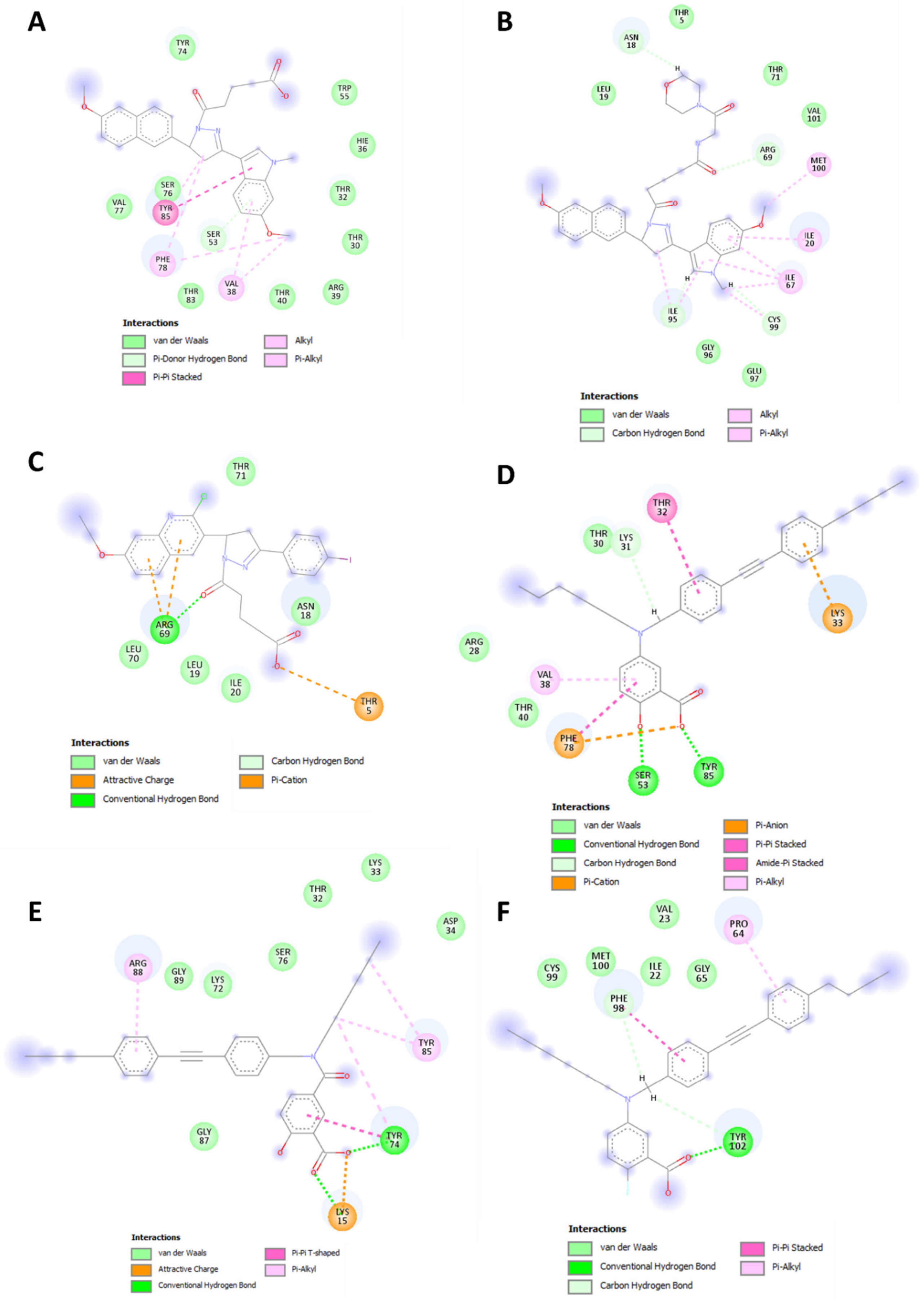

Figure S15: Interaction profile of the top positional cluster of each compound rendered with Discovery Studio v21.1.0.20298. Note that DAZLN-56 (B), TDRL-551 (C) and MS-ML26 (F) do not have any aromatic π-π stacking interactions, while the DAZLN-55 (A), MS-ML24 (D), and MS-ML25 (E) do. These are also the three compounds that bind to the ssDNA binding site of hSSB1. 3D conformational representations of these clusters can be found in Section 1.6 of the supporting information.

1.8 DECOMPOSITION

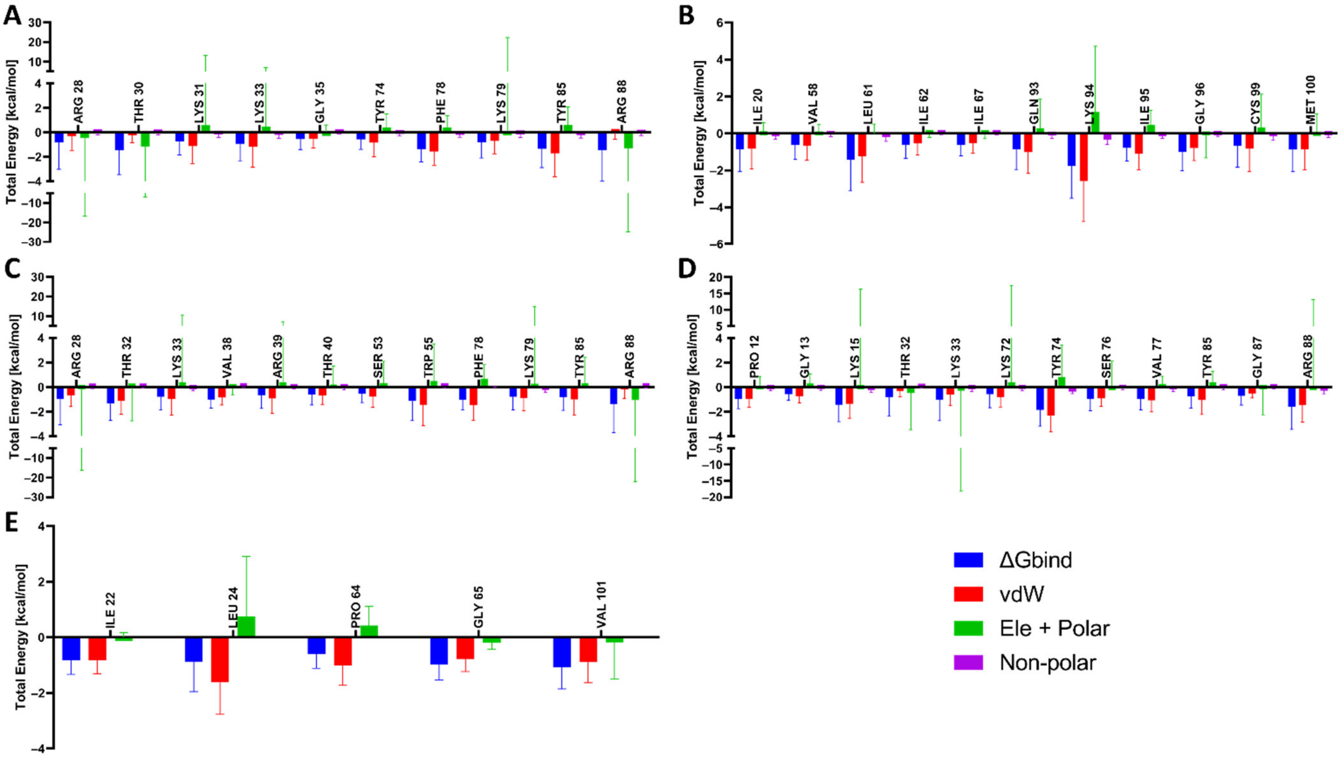

Figure S16: A decomposition of the total pairwise energy (ΔGbind) into van der Waals (vdW), total electrostatic energy (Ele + Pol), and non-polar energy contributions for those lower than –0.5 kcal/mol. (A) DAZLN-55; (B) DAZLN-56; (C) MS-ML24; (D) MS-ML25; (E) MS-ML26.

Supplementary References

1. Richard, D.J.; Bolderson, E.; Cubeddu, L.; Wadsworth, R.I.M.; Savage, K.; Sharma, G.G.; Nicolette, M.L.; Tsvetanov, S.; McIlwraith, M.J.; Pandita, R.K.; et al. Single-Stranded DNA-Binding Protein Is Critical for Genomic Stability. *Nature* **2008**, *453*, 677–681, doi:10.1038/nature06883.
2. Paquet, N.; Adams, M.N.; Ashton, N.W.; Touma, C.; Gamsjaeger, R.; Cubeddu, L.; Leong, V.; Beard, S.; Bolderson, E.; Botting, C.H.; et al. (NABP2/OBFC2B) Is Regulated by Oxidative Stress. *Sci. Rep.* **2016**, *6*, 27446, doi:10.1038/srep27446.
3. Paquet, N.; Adams, M.N.; Leong, V.; Ashton, N.W.; Touma, C.; Gamsjaeger, R.; Cubeddu, L.; Beard, S.; Burgess, J.T.; Bolderson, E.; et al. hSSB1 (NABP2/ OBFC2B) Is Required for the Repair of 8-Oxo-Guanine by the hOGG1-Mediated Base Excision Repair Pathway. *Nucleic Acids Res.* **2015**, *43*, 8817–8829, doi:10.1093/nar/gkv790.
4. Schmidt, D.; Boehm, M.; McClendon, C.L.; Torella, R.; Gohlke, H. Cosolvent-Enhanced Sampling and Unbiased Identification of Cryptic Pockets Suitable for Structure-Based Drug Design. *J. Chem. Theory Comput.* **2019**, *15*, 3331–3343, doi:10.1021/acs.jctc.8b01295.
5. Lawson, T.; El-Kamand, S.; Kariawasam, R.; Richard, D.J.; Cubeddu, L.; Gamsjaeger, R. A Structural Perspective on the Regulation of Human Single-Stranded DNA Binding Protein 1 (hSSB1, OBFC2B) Function in DNA Repair. *Comput Struct Biotechnol J* **2019**, *17*, 441–446, doi:10.1016/j.csbj.2019.03.014.
6. Case, D.A.; Belfon, K.; Ben-Shalom, I.Y.; Brozell, S.R.; Cerutti, D.S.; Cheatham, T.E.; Cruzeiro, V.W.D.; Darden, T.A.; Duke, R.E.; Giambasu, M.K.; et al. AMBER 2020 2020.
7. He, X.; Man, V.H.; Yang, W.; Lee, T.-S.; Wang, J. A Fast and High-Quality Charge Model for the next Generation General AMBER Force Field. *J. Chem. Phys.* **2020**, *153*, 114502, doi:10.1063/5.0019056.
8. Tian, C.; Kasavajhala, K.; Belfon, K.A.A.; Raguette, L.; Huang, H.; Migues, A.N.; Bickel, J.; Wang, Y.; Pincay, J.; Wu, Q.; et al. ff19SB: Amino-Acid-Specific Protein Backbone Parameters Trained against Quantum Mechanics Energy Surfaces in Solution. *J. Chem. Theory Comput.* **2020**, *16*, 528–552, doi:10.1021/acs.jctc.9b00591.
9. Jorgensen, W.L.; Chandrasekhar, J.; Madura, J.D.; Impey, R.W.; Klein, M.L. Comparison of Simple Potential Functions for Simulating Liquid Water. *J. Chem. Phys.* **1983**, *79*, 926–935, doi:10.1063/1.445869.
10. Darden, T.; York, D.; Pedersen, L. Particle Mesh Ewald: An N·log(N) Method for Ewald Sums in Large Systems. *J. Chem. Phys.* **1993**, *98*, 10089–10092, doi:10.1063/1.464397.
11. Lee, T.-S.; Allen, B.K.; Giese, T.J.; Guo, Z.; Li, P.; Lin, C.; McGee, T.D.; Pearlman, D.A.; Radak, B.K.; Tao, Y.; et al. Alchemical Binding Free Energy Calculations in AMBER20: Advances and Best Practices for Drug Discovery. *J Chem Inf Model* **2020**, *60*, 5595–5623, doi:10.1021/acs.jcim.0c00613.
12. Mermelstein, D.J.; Lin, C.; Nelson, G.; Kretsch, R.; McCammon, J.A.; Walker, R.C. Fast and Flexible Gpu Accelerated Binding Free Energy Calculations within the Amber Molecular Dynamics Package. *Journal of Computational Chemistry* **2018**, *39*, 1354–1358, doi:10.1002/jcc.25187.

13. Roe, D.R.; Cheatham, T.E.I. PTRAJ and CPPTRAJ: Software for Processing and Analysis of Molecular Dynamics Trajectory Data. *J. Chem. Theory Comput.* **2013**, *9*, 3084–3095, doi:10.1021/ct400341p.

14. Goddard, T.D.; Huang, C.C.; Meng, E.C.; Pettersen, E.F.; Couch, G.S.; Morris, J.H.; Ferrin, T.E. UCSF ChimeraX: Meeting Modern Challenges in Visualization and Analysis. *Protein Sci.* **2018**, *27*, 14–25, doi:10.1002/pro.3235.

15. Pettersen, E.F.; Goddard, T.D.; Huang, C.C.; Meng, E.C.; Couch, G.S.; Croll, T.I.; Morris, J.H.; Ferrin, T.E. UCSF ChimeraX: Structure Visualization for Researchers, Educators, and Developers. *Protein Sci.* **2021**, *30*, 70–82, doi:10.1002/pro.3943.

16. BIOVIA Discovery Studio Visualizer Version 21.0.0 2022.

17. Srinivasan, J.; Cheatham, T.E.; Cieplak, P.; Kollman, P.A.; Case, D.A. Continuum Solvent Studies of the Stability of DNA, RNA, and Phosphoramidate−DNA Helices. *Journal of the American Chemical Society* **1998**, *120*, 9401–9409, doi:10.1021/ja981844+.

18. Genheden, S.; Ryde, U. The MM/PBSA and MM/GBSA Methods to Estimate Ligand-Binding Affinities. *Expert Opin Drug Discov* **2015**, *10*, 449–461, doi:10.1517/17460441.2015.1032936.

19. Fanning, E.; Klimovich, V.; Nager, A.R. A Dynamic Model for Replication Protein A (RPA) Function in DNA Processing Pathways. *Nucleic Acids Res.* **2006**, *34*, 4126–4137, doi:10.1093/nar/gkl550.

20. Ren, W.; Chen, H.; Sun, Q.; Tang, X.; Lim, S. choo; Huang, J.; Song, H. Structural Basis of SOSS1 Complex Assembly and Recognition of ssDNA. *Cell Rep.* **2014**, *6*, 982–991, doi:10.1016/j.celrep.2014.02.020.

21. Bochkarev, A.; Pfuetzner, R.A.; Edwards, A.M.; Frappier, L. Structure of the Single-Stranded-DNA-Binding Domain of Replication Protein A Bound to DNA. *Nature* **1997**, *385*, 176–181, doi:10.1038/385176a0.

22. Cheeseright, T.; Mackey, M.; Rose, S.; Vinter, A. Molecular Field Extrema as Descriptors of Biological Activity: Definition and Validation. *J Chem Inf Model* **2006**, *46*, 665–676, doi:10.1021/ci050357s.

23. Kuhn, M.; Firth-Clark, S.; Tosco, P.; Mey, A.S.J.S.; Mackey, M.; Michel, J. Assessment of Binding Affinity via Alchemical Free-Energy Calculations. *J Chem Inf Model* **2020**, *60*, 3120–3130, doi:10.1021/acs.jcim.0c00165.

24. Daina, A.; Michielin, O.; Zoete, V. SwissADME: A Free Web Tool to Evaluate Pharmacokinetics, Drug-Likeness and Medicinal Chemistry Friendliness of Small Molecules. *Sci Rep* **2017**, *7*, 42717, doi:10.1038/srep42717.
